# Future Premature Mortality Due to O_3_, Secondary Inorganic Aerosols and Primary PM in Europe — Sensitivity to Changes in Climate, Anthropogenic Emissions, Population and Building Stock

**DOI:** 10.3390/ijerph120302837

**Published:** 2015-03-04

**Authors:** Camilla Geels, Camilla Andersson, Otto Hänninen, Anne Sofie Lansø, Per E. Schwarze, Carsten Ambelas Skjøth, Jørgen Brandt

**Affiliations:** 1 Department of Environmental Science, Aarhus University, Frederiksborgvej 399, P.O. Box. 358, 4000 Roskilde, Denmark; E-Mails: asla@envs.au.dk (A.S.L.); jbr@envs.au.dk (J.B.); 2 Swedish Meteorological and Hydrological Institute, Norrköping SE-60176, Sweden; E-Mail: camilla.andersson@smhi.se; 3 Department of Health Protection, National Institute for Health and Welfare (THL), 70701 Kuopio, Finland; E-Mail: otto.hanninen@thl.fi; 4 Department of Air Pollution and Noise, Norwegian Institute of Public Health, 4404 Nydalen, 0403 Oslo, Norway; E-Mail: per.schwarze@fhi.no; 5 National Pollen and Aerobiological Research Unit, Institute of Science and the Environment, University of Worcester, Worcester WR2 6AJ, UK; E-Mail: c.skjoth@worc.ac.uk

**Keywords:** integrated assessments, air pollution, health effects, climate change, future anthropogenic emissions, population developments, infiltration

## Abstract

Air pollution is an important environmental factor associated with health impacts in Europe and considerable resources are used to reduce exposure to air pollution through emission reductions. These reductions will have non-linear effects on exposure due, e.g., to interactions between climate and atmospheric chemistry. By using an integrated assessment model, we quantify the effect of changes in climate, emissions and population demography on exposure and health impacts in Europe. The sensitivity to the changes is assessed by investigating the differences between the decades 2000–2009, 2050–2059 and 2080–2089. We focus on the number of premature deaths related to atmospheric ozone, Secondary Inorganic Aerosols and primary PM. For the Nordic region we furthermore include a projection on how population exposure might develop due to changes in building stock with increased energy efficiency. Reductions in emissions cause a large significant decrease in mortality, while climate effects on chemistry and emissions only affects premature mortality by a few percent. Changes in population demography lead to a larger relative increase in chronic mortality than the relative increase in population. Finally, the projected changes in building stock and infiltration rates in the Nordic indicate that this factor may be very important for assessments of population exposure in the future.

## 1. Introduction

Air pollution is a major environmental factor associated with health impacts [1,2]. Air pollution and its impacts on environment and human health are efficiently studied by integrated assessment systems. These integrated systems connect models from various scientific disciplines (e.g., atmospheric science, epidemiology, public health and economics). In Europe, the integrated Greenhouse gas and Air pollution INteractions and Synergies (GAINS) system is used as a basis for emission control strategies. GAINS can be used to identify cost-effective strategies for limiting air pollution and the related negative effects [3]. Integrated systems that operate on sub-national to European scales include the UK Integrated Assessment Model (UKIAM, [4]) and the Danish Economic Valuation of Air pollution (EVA [5]) system. Such integrated systems can be used to project how impacts and associated costs changes in relation to existing or future air pollution levels. This is done by including scenarios for climate change and for the development in emissions of air pollutants in combination with a full simulation with a Chemical Transport Model (CTM) or with standardized source-receptor relationships (calculated with a CTM). The use of source-receptor relationships has the advantage of reducing the computing time significantly and has therefore been used in policy tools like GAINS. However, in order to better account for non-linear chemistry-transport processes in the atmosphere [6] and the possible impact from climate change on e.g., biogenic emissions and atmospheric processes, a full CTM simulation needs to be carried out. Studies of the future development in atmospheric pollution levels due to climate and emission changes have mainly focused on ozone (O_3_) and particulate matter (PM) (e.g., [7,8,9,10]). O_3_ is gas formed in the atmosphere through a number of chemical reactions related to both anthropogenic and natural precursor emissions. Due to a long atmospheric life time O_3_ can be transported over large distances in the atmosphere. PM is composed of both primary components emitted directly from the source and secondary inorganic (SIA) and organic (SOA) aerosols formed in the atmosphere. The life time of the different PM components lies within hours to weeks. Both O_3_ and PM are linked to cardiovascular and respiratory premature mortality [11,12,13]. More recently it has also been documented that exposure to nitrogen dioxide (NO_2_) is associated to significant negative health effects [12,13,14]. NO_2_ is both emitted directly and formed in the atmosphere due to emissions from e.g. traffic and the highest levels are observed close to major roads [13]. NO_2_ and O_3_ are together with volatile organic compounds (VOC) linked through non-linear photochemistry in the atmosphere. 

The overall exposure to atmospheric pollutants can be assessed by combining the estimated surface concentrations with gridded demography and corresponding concentration-response functions. In the current study we only focus on the health effects related to O_3_, primary emitted PM_2.5_ (as Black Carbon (BC) and Organic Carbon (OC) from anthropogenic sources) and the SIA-components of particulate matter. It was not possible to include SOA, since the complex chemistry describing the formation of secondary organic aerosols was not included in the two CTMs when the study was conducted. Furthermore, concentration-response functions for NO_2_ have not been included, since they have been found to be too uncertain for a regional scale assessment like the present. 

At the European scale the expected change in the demography [15] can alter both the exposure and the sensitivity towards air pollution e.g., due to either urbanization and a general trend towards an “ageing society”. An aging population may be more sensitive to the exposure of air pollution [16,17], which means that robust projections on premature mortality due to air pollution must take into account both changes in demography as well as changes in emissions and climate. The estimates for present day conditions vary substantially due to differences in methods and underlying input data. An ensemble of CTM models estimated the global premature mortality associated with O_3_ to range from 140,000 to 900,000 deaths per year (with a multi-model mean of 470,000) [18]. PM_2.5_-related diseases were estimated to cause between 1.3 and 3.0 million deaths per year (mean of 2.1 million). They also included an uncertainty range on the applied concentration-response functions and conclude that this uncertainty dominates the overall uncertainty, but differences in modelled concentrations also add to the large spread in the mortality estimate. At the European scale, multi-model studies with CTMs have shown variations in the simulated concentrations of O_3_ and PM components, both for present day conditions and for future projections due to differences in the chemical schemes and parameterizations used, but also due to differences in sensitivities to climate change [10,19]. The impact of climate change on ozone related mortality and morbidity in Europe was recently investigated by Orru*, et al.* [20] using the climate projections A2 and A1B. They found an increased overall hospitalization and mortality for both scenarios with the more pessimistic A2 scenario resulting in a 40% larger increase compared to the A1B scenario. The overall projections of the development in climate have, within the IPCC assessments, shown a large spread even when the same emissions scenario are used in the climate models [21]. These differences are large enough to impact the projections of future air pollution levels using CTM models [22]. A multi-model approach including several climate change realizations and several CTMs can therefore be used to explore the full a range of air pollution scenarios and the related premature deaths. Likewise, single-model studies are useful to illustrate one out of many possible future developments in air pollution levels and to identify governing processes and explore the sensitivity to key drivers and associated health effects. Worldwide there are very few such studies that focus on the sensitivity to underlying modeling choices [23].

The main purpose of this study is to make an assessment of premature deaths due to future air pollution levels and to investigate how sensitive the assessment is to input data and assumptions such as emission reductions and climate change scenarios. The Danish Economic Valuation of Air pollution (EVA) model system [5] is used in combination with two climate models and two CTM models. We test how sensitive the final health outcome is to changes in the main drivers defined as climate, anthropogenic emissions and population data, by changing these stepwise. Furthermore, also the combined effect of all data sets will be analysed. The assessment is done for all of Europe with special focus on the Nordic region. The results with the EVA system are based on three decadal long simulations for the 2000s (2000–2009), 2050s (2050–2059) and 2080s (2080–2089). The EVA system is based on exposure-response functions derived primarily from large cohort and epidemiological studies in mainly Europe and the U.S. (e.g., [24,25]). The actual doses that the population are exposed to will in reality depend on a number of factors that are difficult to include in larger scale assessments like the current, e.g., the actual micro-environmental concentrations (personal exposures), physical activity (breathing rate) and, in the case of PM, particle size distribution and possibly the chemical composition. Also the infiltration rate into the houses will be an important factor for the actual exposure. The current study will therefore attempt to include changes in the infiltration rate due to future changes in the building stock. For this part the focus will be on the Nordic region where the Nordic Energy Performance of Buildings (EPB) Directive (2010), requires that all new buildings by 2020 will comply with nearly zero energy standards. We will include an evaluation of how such changes in the building stocks with increased tightening of the buildings will affect the infiltration rate and hence the doses of air pollution that the Nordic population are exposed to. The total exposure will also depend on indoor sources like e.g., fire places that are widely used in Scandinavia. In addition to this combustion component there are numerous components, more or less specific to indoor air, that are not included in the present assessment. 

The structure of the paper is as follows: in Section 2 a general overview of the study and the input data is given, including a more detailed description of the individual models. In Section 3 the results of the stepwise change in input data are presented and discussed. Conclusions are summarized in Section 4. 

## 2. Study Setup

The basis of the study is a model chain that previously has been used for studies of the future development of the ozone concentration and nitrogen deposition in Europe [10,19]. In the current study the model chain consists of the two CTMs DEHM and MATCH that cover the Northern hemisphere and Europe with a resolution of 150 km × 150 km and 50 km × 50 km, respectively. The hemispheric model DEHM provides boundary conditions to the regional model MATCH, whereby the inflow from non-European emission sources is included. The same set of anthropogenic emissions and global climate scenario has been used as input to the two models. However, in the higher resolution model MATCH the applied climate data from the ECHAM model has been dynamically downscaled through the regional climate model RCA3. 

**Figure 1:**
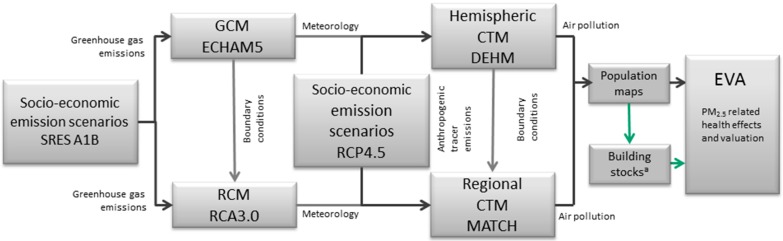
An illustration of the model chain and the required input data. The green arrows indicate the new terms related to the building stocks and infiltration. **^a^** Tentatively accounted for here—for the first time in relation to climate change exposure scenarios.

In the current study the model chain has been extended to include the EVA system. Thereby the concentration levels of various air pollutants are converted to human exposure and related health impacts. This new model chain is illustrated in Figure 1. The full model chain has been run for three decades, 2000–2009, 2050–2059 and 2080–2089, in order to reflect the conditions during present day, mid-century (near future) and at the end of the century (distant future). The final results are presented as decadal means. Information about the models and the necessary input data are summarized in the following subsections.

### 2.1. The DEHM Model

The Danish Eulerian Hemispheric Model (DEHM) is an offline Eulerian CTM covering the Northern Hemisphere and with a nesting procedure allowing for several nests with higher resolution over Europe, Northern Europe and Denmark [26,27,28]. In the current setup only the main domain of the Northern Hemisphere with a horizontal resolution of 150 km × 150 km and 20 vertical layers has been used, with the top of the model defined at 100 hPa. DEHM has been used in a number of studies to investigate the future developments in air quality due to changes in emission and/or climate [7,10,19,29,30,31,32,33]. 

The current model version simulates concentration fields of 58 photo-chemical compounds (including NO_x_, O_3_, SO_x_, VOC, NH_x_, CO, *etc*.) and nine classes of particulate matter (e.g., PM_2.5_, PM_10_, TSP, sea-salt < 2.5 µm, sea-salt > 2.5 µm, Black Carbon (BC), and fresh and aged Organic Carbon (OC)). These are described by 122 chemical reactions. Natural emissions of NOx from lightning/soil and NH_3_ from vegetation/soil are based on the Global Emission Inventory Activity (GEIA); while natural emissions of sea salt [34,35] and biogenic isoprene [36] are calculated on-line in DEHM as a function of meteorological parameters e.g., temperature and wind speed and they are thereby allowed to change according to changes in climate.

Meteorological parameters for the present day and future conditions are for DEHM obtained from the coupled global atmosphere-ocean model ECHAM5/MPIOM. The projections are based on the SRES A1B emission scenario [37] made as part of the IPCC-AR4 model intercomparison [38]. Previously, DEHM was coupled to the ECHAM4 model and a detailed evaluation showed a reasonable agreement between simulated and observed present day levels of a number of air pollutants [30]. However, in the current setup with the ECHAM5/MPIOM A1B (run 3) simulations, an evaluation of the N-components has indicated an underestimation of the atmospheric mixing heights over parts of Europe [19]. Therefore a climatology of the mixing heights has been constructed based on a 10 year (2000–2009) simulation with the meteorological model MM5v3 [39]. In the current DEHM-ECHAM setup the original mixing heights have been replaced by this climatology for both the present days and future periods.

### 2.2. The MATCH Model

Multi-scale Atmospheric Transport and Chemistry (MATCH [40]) is an offline, Eulerian regional scale CTM that has previously been used to simulate the effect of climate and/or emission change on surface ozone (e.g., [9,41,42,43]), nitrogen deposition (e.g., [19,44,45,46]) and particle concentration [47]. The MATCH model includes ca. 70 chemical species and ca. 130 photochemical, thermal and wet phase chemical reactions. The chemical scheme describes ozone forming photochemistry and the formation of secondary inorganic aerosol species. Other particle species (primary BC, OM and sea salt) are described with a bulk model with four size bins. Secondary organic aerosol and natural dust particles are not included in the current model version. Biogenic isoprene emissions [48,49] and sea salt emissions [50] from saline water surfaces were dependent on the meteorological situation and thus impacted by climate change. Dry deposition of reactive gases was simulated dependent on relative humidity and soil moisture as described in [9]. Europe is covered by a horizontal resolution of 50 km × 50 km and 13 vertical levels reaching ca. 5 km height. Concentrations of a number of chemical species from the DEHM model are read every six hours at the lateral and upper boundaries of the MATCH model.

Meteorological parameters for MATCH are simulated based on the global climate realization that is used also for the DEHM simulations and the greenhouse gas emission projection is the same, SRES A1B [37]. The meteorological data from ECHAM5/MPIOM is used as boundaries in a regional climate model, the Rossby Centre Regional Climate model version 3 (RCA3). Model description and evaluation of both current and future climate simulated with RCA3 is given by [51,52]. The climate projection used here is the downscaling named ECHAM5 A1B-r3 [52]. Six-hourly meteorological outputs at 21 model levels and a range of output variables at the surface were used in the ofﬂine MATCH modelling. 

The climate as downscaled by RCA3 carries on broad features of the climate simulated by the parent GCM. The climate projection used here has a temperature increase until the period 2041–2070 close to the average of an ensemble of 16 different projections downscaled from different GCM runs by RCA3 over Europe [52]. Apart from increases in temperature, the climate projection shows an increase in summer precipitation in large parts of northern Europe extending also over Poland and Germany, while precipitation generally decrease in southern Europe. 

### 2.3. The Anthropogenic Emissions

Both CTM models use the same anthropogenic emission scenario of air pollutant precursors: primary PM_2.5_ (OC, BC), and primary gaseous components (nitrogen oxides (NO_x_), sulphur oxides (SO_x_), non-methane hydrocarbons (NMVOC) and ammonia (NH_3_)) from the RCP4.5 emission scenarios. This emission scenario is part of the so-called Representative Concentration Pathways (RCPs), which describe the developments in emissions as used in the fifth IPCC Assessment Report (AR5) [53]. The RCP4.5 scenario was constructed to give a “medium” climate impact (an anthropogenic radiative forcing equal to 4.5 W/m^2^) towards the end of the century. The assumed changes in emissions towards the 2050s are in general lower for most components than a “current legislation” scenario produced by IIASA for the ECLIPSE/ECLAIRE projects [19]. An exception is NH_3_ where the RCP4.5 assumes a small decrease towards 2050, while the IIASA scenario includes a very small increase. For the three periods in focus here the RCP4.5 emissions for 2000, 2050 and 2080 have been used including standard temporal profiles for the variation on diurnal to seasonal timescales. Reductions in the range of 50% to 85% are projected for most components towards 2080. An exception is ammonia, for which the total reduction is much smaller (23% in 2080). These numbers are for Europe in total, but at the regional scale the reductions can be smaller or larger. The emissions for 2000, 2050 and 2080 can be seen in Table 1. 

**Table 1:** RCP4.5 emissions (Tg) as total for the European domain for 2000, 2050 and 2080. The changes in % relative to the emissions in 2000 are also given. The numbers marked with red corresponds to the additional 2080b scenario for ammonia.

Year	SO_x_ [Tg]	Change [%]	NO_x_ [Tg]	Change [%]	NMVOC [Tg]	Change [%]	Primary PM_2.5_/[Tg]	Change [%]	NH_3_ [Tg]	Change [%]
2000	9.6		7.3		19.7		2.7		6.4	
2050	2.8	−71	3.8	−48	13.5	−31	1.5	−43	5.8	−9
2080	1.4	−85	2.6	−64	9.6	−51	0.8	−69	5.0	−23
2080b									6.7	+4

Recent studies have concluded that the projected temperature increase in Europe can lead to an increase of the ammonia emissions in the agricultural sector of up to 40% towards the end of the century [54,55]. We have therefore included an additional emission scenario, where the RCP4.5 ammonia emission for 2080 was increased by 35% (called scenario 2080b). All in all this gives a 4% increase in the 2080 ammonia emissions relative to present day (see the numbers in red in Table 1). By including this scenario, we can evaluate whether a climate penalty on ammonia emissions can alter the health effect related to air pollution in the future. 

### 2.4. The EVA System

The integrated model system, Economic Valuation of Air pollution (EVA) has been developed to quantify and assess health impacts as well as health-related economic externalities of air pollution. The EVA system has previously been used to assess the total impacts on human health and related external costs due to total air pollution levels in Europe and Denmark [5,56]. 

The EVA system is based on the impact pathway chain. In this study, the EVA system integrates output from the regional-scale chemistry transport models, (DEHM and MATCH), gridded population data, and exposure-response functions for health impacts. By using the comprehensive and thoroughly tested chemical transport models, the EVA system takes into account the non-linear atmospheric processes induced from specific changes in climate on air pollution levels. Further, the geographic domain used in DEHM covering the Northern Hemisphere allows for a description of the intercontinental atmospheric transport of pollutants into Europe. 

Calculated concentrations are combined with gridded population data for Europe, to calculate the exposure. Population-level health outcomes are estimated by combining the population-level exposure with a number of exposure-response functions (ERF), including different morbidity outcomes as well as short-term (acute) and long-term (chronic) mortality, related to exposure of ozone and SO_2_ (short-term) and PM_2.5_ (long-term), respectively. Furthermore, infant mortality is included for exposure of PM_2.5_. The morbidity outcomes include chronic bronchitis, restricted activity days, congestive heart failure, lung cancer, respiratory and cerebrovascular hospital admissions, asthmatic children (<15 years) and adults (>15 years), which includes bronchodilator use, cough, and lower respiratory symptoms. The exposure-response functions all have the form given in Equation (1):

(1) R=α·δc·P

where *R* is the response (e.g., in cases, days, or episodes), *δc* is the delta-concentration (*i.e*., the additional concentration resulting from emissions of a particular emission source) or in this case the total concentration levels, *P* is the affected share of the population, and *α* is a constant for the particular health outcome, typically based on empirically-determined relative risks collected from registries and obtained from published cohort studies. See Brandt *et al.* (2013) [5] for a detailed list of the ERFs. 

Relevant chemical compounds (*i.e.*, those endorsed by the WHO and the EU Commission) are included in the study. For compounds in aerosol phase, the impacts are assumed to be proportional to their contribution to the particle mass of PM_2.5_. Presently, the compounds related to human health impacts included in the EVA system are: O_3_, CO, SO_2_, SO_4_^2−^, NO_3_^−^, and the primary emitted part of PM_2.5_. For the secondary inorganic components, the full weight of the particles including nitrate, sulphate and ammonium is applied as follows: Besides SO_4_^2−^ the full weight of H_2_SO_4_, NH_4_SO_4_ and (NH_4_)_2_SO_4_ are included. The same applies for NO_3_^-^ where also NH_4_NO_3_ is included. The primary particles include BC and OC (fresh and aged). Secondary organic particles are not yet implemented in the calculation by the EVA system. 

### 2.5. Population Data

An important input to the EVA system is information on the distribution and age of the population across Europe. These data are needed on a spatial grid for the three focus periods. As part of the European projects INTARESE and HEIMTSA a number of gridded population data sets have previously been constructed, based on various data sources ranging from local administration units (LAU-level 2) to UN data on country level (for details see [57]). From these projects population data sets for the year of 2000 and projections for 2050 have been obtained. Population data for 2080 was not available from this dataset; the sensitivity analysis was therefore only based on simulations for the two decades 2000s and 2050s. In the projections several growth rates were applied, but it is recommended to use the so called “medium” rate for health assessments, thus this data set is used for 2050. The data covers 30 countries in Europe on a 50 km × 50 km grid and includes information on the age structure of the population (see Figure S2a in the Supplement Materialfor the geographical distribution in 2000). The applied age structure for 2000 and 2050 is shown in Figure S1 for all of Europe and for the Nordic region alone. 

### 2.6. The Building Stock

Population based studies in Europe have shown that PM_2.5_ infiltration of the outdoor PM_2.5_ levels in the existing building stock typically varies from 50%–90% [58]. Comparisons of buildings from Northern, Central and Southern European climatological regions show an increasing trend in infiltration in warmer climates [59]. The European Directive on Energy Performance of Buildings [60] requires that all new buildings by 2020 will comply with nearly zero energy standards. In practice, especially in Nordic countries, this requires heat recovery technology and therefore mechanical ventilation systems. More detailed analysis of infiltration in buildings using different ventilation systems in Helsinki has demonstrated an effect of the filtration in mechanical ventilation systems [61]. Further, the infiltration rate is strongly dependent on the particle size distribution [62]. In old buildings mechanical ventilation systems are not yet common, but as the buildings are renovated, the coverage of mechanical ventilation systems with efficient filtration is constantly increasing.

The development of the European building stock is guided by the EU Energy Roadmap [63], stating that the EU is committed to reduce greenhouse gas emissions to 80%–95 % below 1990 levels by 2050. Higher energy efficiency in new and existing buildings is identified as the key to reach this target, making nearly zero-energy buildings the norm by 2050. According to Erhorn-Kluttig and colleagues [64] this will require building air tightness values below 1 Air Changes per Hour at 50 hPa (ACH50 = 1.0 h^−1^). The current work assumes that such tightness numbers will be reached by 2050 and will be sustained from thereon, resulting in the PM_2.5_ infiltration rates shown in Table 2. The estimates are based on a building stock renewal rate of 2% a^−1^, and a measured PM_2.5_ infiltration equal to 59% in year 2000 and increasing air tightness of buildings from ACH50 = 8 to ACH50 = 1 by 2050. It should be noted that other impacts of tighter houses (e.g., possible increase of molds) as well as the size distribution of the particles are not considered here. 

**Table 2:** Projected PM_2.5_ infiltration and air tightness of buildings in Nordic countries.

Decade	Building Tightness ^a^ ACH50	PM_2.5_ Infiltration	References
2000	5	59%	Hänninen *et al*. 2004, 2011, 2013 [58,59,62], Kearney *et al*. 2014 [65], Gens *et al*., 2014 [66], Zou, 2010 [67],
2020	3	52%	EC 2012 [63],
2050	1	30%	Erhorn-Kluttig *et al*., 2009 [64].
2080	1	24%	
2100	1	21%	

^a^ average ACH50 in new buildings.

### 2.7. Limitations and Uncertainties

Before discussing the results it is important to underline the limitations and uncertainties related to the applied methodology. The main uncertainties arise from lacking species in the impact assessment, the coarse spatial resolution and the exposure-response functions that were used. Organic aerosols (OA) include both primary (POM) and secondary (SOA) components, and constitute a large part (20%–90%) of the measured PM_2.5._ Due to the complex nature of the sources and of the oxidation-processes leading to SOA, modelling of OA is still very uncertain [21,68,69]. In this study we investigate only the health impacts of the PM_2.5_ components: primary BC and OC, and SIA. We chose to exclude natural components (e.g., sea salt and desert dust) since we are interested in the impact of anthropogenic activities. We also omit SOA, since the models included did not describe this complex chemistry at the time when the study was conducted. The total anthropogenic PM_2.5_ concentration simulated by the models will therefore be underestimated, and hence will the resulting health effects also be underestimated. When SOA recently was included in the GAINS system, it was concluded that the estimated negative health impacts related to PM_2.5_ in Europe in year 2000 increased by 19% [70]. This includes both anthropogenic and biogenic SOA. The biogenic fraction of SOA is modelled to be around 20%, but the estimation of biogenic emissions is very uncertain and can vary by a factor of five [10]. Based on this we conclude that for current day conditions the models in this study underestimate the health impact of PM_2.5_ by approximately 19% due to lack of SOA, but this estimate is uncertain. BVOC emissions and hence the production of surface O_3_ and biogenic SOA have been projected to increase in the future due to warming [71]. But in recent studies this sensitivity is reduced due to a CO_2_ inhibition effect on the natural emissions and the land-use changes is now considered a key factor for the development of e.g., SOA [72]. Changes to temperature and oxidative capacity may also alter the SOA concentration in the future. We refrain from speculation on how the omission of SOA impact our future estimates, and leave such a study for when the models are sophisticated enough to allow a thorough investigation thereof. Our estimates of change in impact of PM_2.5_ focus on the change in anthropogenic primary PM components and SIA. 

The simulation of the included gaseous and particulate species is likewise associated with uncertainties that can introduce a positive or negative bias into the analysis depending on the chemical component. The evaluation in Section 3.1 gives an overview of the ability of the two models to capture current day levels of the primary and secondary components included here. The relatively low resolution in the models and input data adds to the uncertainty of the simulated air quality levels and the exposure. Previous studies have shown that a higher (10–20 km) resolution is important for modelling of the urban signal of NO_2_, but less important for secondary components like SIA, SOA and O_3_ [73,74]. Nevertheless, exposure to primary PM concentrations originating from local sources will be underestimated with the resolution used in the two CTMs in the current study. Exposure to O_3_ will on the other hand be overestimated. It is outside the scope of this study to estimate the health impact of urban or local scale concentrations; we focus on the regional scale impacts. 

The use of a relatively coarse resolution is a tradeoff in order to simulate three full decades. Due to interannual variability it is necessary to cover such a long time period. Running the models for a full decade and making an average afterwards secures that the results are not representing a single extreme year. This could be the case if only one or a few meteorological years were used. Further the 10-year periods were chosen carefully in order to avoid influence of decadal variability. As the focus here is on the changes between the decades, it is important to test if the change is significant. In the following a student’s t-test is used to test whether the change in e.g., chronic mortality between the 2000s and the 2050s is significant compared to year-to-year variations within the decades. Even if there is a large uncertainty related to these projections, this is still a useful method to investigate if a single set of input data (e.g., the climate or population data) have an important impact on the assessment.

The health impact assessment will be very sensitive to the exposure-response (ER) functions in the EVA system, where e.g., a linear function between PM_2.5_ concentration and health effects has been chosen (see Equation (1)). We have been rather conservative concerning the implementation of exposure-response functions to only include functions that are approved by the WHO. This is important when using the results for decision support, since e.g., any tendency towards an exaggeration of health effects due to uncertain concentration-response function, will decrease the receptivity of decision makers to modelled results. Concerning NO_2_, the exposure-response functions have been found to be too uncertain, and it is difficult to distinguish long-term effects to exposure in the rural and urban background from short-term effects from episodes in street canyons in cities where the NO_2_ concentrations are much higher. Therefore, we have chosen not to include effects from NO_2_ before more evidence on the impacts and the possibility to distinguish short-term from long-term effects have been found. There is increasing knowledge on the toxicity of the individual particle components and it has been argued that combustion particle like BC could be used as an additional indicator in assessments [75]. However, a recent review initiated by the World Health Organization concludes that “assessments based on PM_2.5_ studies will be the most inclusive” as the data on e.g., BC and the related health outcome is less comprehensive [13]. The other indicators can be used for sensitivity analysis and this has previously been done with the EVA system to assess the impact of assuming that primary particles (including BC, OC and mineral dust) are more harmful to human health compared to the average PM_2.5_ [5]. In the current assessment we have, however, focused on the sensitivity to some of the other main model assumptions/drivers. 

Current ER functions are based on outdoor concentrations. As the infiltration changes to the future these functions could change. For the projections it is assumed that the outdoor exposure-response relationship remains constant over the whole century. The impact of changes in the building stocks has been estimated separately with its own inherent uncertainties (Section 3.5), but this does not account for e.g., any changes in population time-activity, or changes in the fraction of time spent in suburban and centralized city regions. E.g. policies tightening the urban structures would lead to higher exposures due to higher correlation between population density and emissions. The disease burden of the population could also change in the future leading to more/less sensitivity to air pollution. So far knowledge and/or data are not sufficient to cover these issues in a regional scale and long-term projection as the current. Recent estimates suggest that PM_2.5_ from outdoor air represents roughly 50% of the burden of disease associated with indoor exposures [76]. Future indoor exposures are likely to change substantially from what they are today. The current work focuses strictly on outdoor pollution and therefore we cannot give any quantitative estimates on the relative role of indoor sources over the target period (2000–2080).

The use of meteorology from climate models to force CTMs as compared to using analyzed meteorology adds to the uncertainty of the simulated O_3_ and PM values [22], but there is no other way to simulate future climate conditions. As described above the two CTMs are driven by the same overall climate projection, but the MATCH model uses a dataset that has been downscaled through the RCA3 climate model, while the DEHM models uses the lower resolution ECHAM-5 dataset directly. Earlier studies using the same setup have shown that part of the differences seen in the simulated air pollution fields by DEHM and MATCH can be linked to differences in both resolution and projected change in the meteorological parameters [10]. Even if the broad features of the temperature distribution are the same, a somewhat warmer climate is projected over Europe by ECHAM-5 than by RCA3. In terms of precipitation ECHAM-5 in general estimates more rainfall than RCA3 over large parts of Europe except in a few regions like western Norway and the Alps. Overall, the projected changes in precipitation across Europe are similar in ECHMA-5 and RCA3. 

Finally, the use of a single climate scenario and a single projection of the development in anthropogenic emissions and the demography will be very determining for the results. For example we have chosen to apply the RCP4.5 emission scenario for air pollutant precursor emissions, which is a so-called “intermediate mitigation” scenario. Use of a business as usual (RCP8.5) scenario or a scenario that aim to limit the increase in global temperature to 2 °C (RCP2.5) could for some components have large impacts on the assumed emissions towards the end of the century [77]. 

## 3. Results and Discussion

The overall objective of this study is to assess whether projected future changes in climate, emissions and population will have a significant effect on air pollution related human health impacts. We therefore use the integrated model chain to test how sensitive the final health outcome is to changes in the main input data, by changing these stepwise. The robustness of the results is also assessed by including air pollution simulations made with the two different CTMs: DEHM and MATCH.

### 3.1. Base Case

The basis for the sensitivity analysis is model simulations for the period 2000–2009 (now called 2000s) with meteorology representing the 2000s as well as anthropogenic emission and population data for year 2000. An evaluation against measurement data from the EMEP network shows differences between the two CTMs: the secondary inorganic aerosol species are generally overestimated by DEHM (17%–88%), whereas MATCH has a lower bias for these (−9%–8%).Both models underestimate EC and OC. The spatial Pearson correlation coefficient obtained was similar between the models for the secondary species, being lowest for reduced nitrogen (ca 0.47) and highest for sulphate (ca 0.78). For the primary particles the spatial Pearson correlation coefficient was higher in MATCH (0.90 and 0.62 for EC and OC respectively) than in DEHM (0.70 and 0.50 respectively). This is expected due to the coarser resolution of DEHM compared to MATCH. Ozone was evaluated in the earlier study with a similar setup, showing a reasonably good agreement with measurements across Europe [10]. 

**Figure 2:**
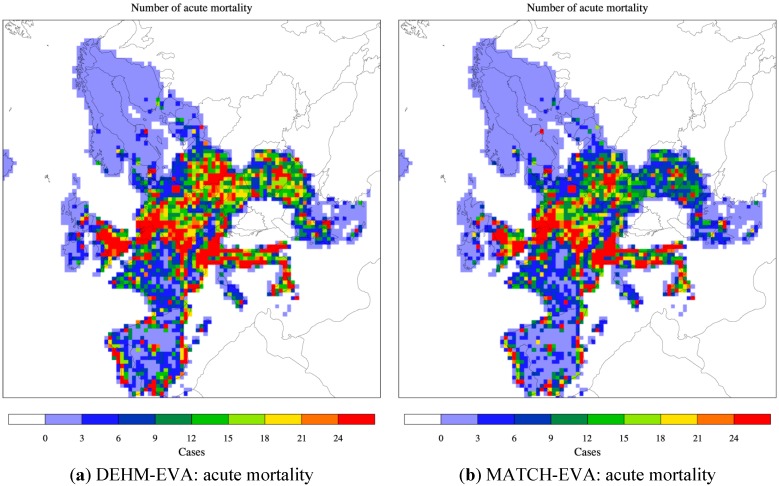
The spatial distribution of acute mortality across Europe related to O_3_ (SOMO35). Based on DEHM (**a**) and MATCH (**b**) simulations with climate, emissions and population data for 2000 (the base “present day” case). The area of a grid cell is 50 km × 50 km = 2500 km^2^. The colour bar refers to number of cases per year per 2500 km^2^. See Figure 4 and Figure 6 for the total number for Europe and the Nordic area.

The resulting yearly surface fields as simulated by the two CTMs have been used as input to the EVA system. The impact from PM is based on the simulated fields of primary PM_2.5_, NO_3_^−^ and SO_4_^2−^ as well as NH_4_^+^ when this component is bound to sulphate and nitrate. The impact from ozone is based on Sum of Ozone Means Over 35 ppb (SOMO35), the sum of the daily maximum of the running 8-hour mean ozone above 35 ppb. Finally also fields of SO_2_ are included. The results from DEHM, MATCH and the used population data have all been converted to a common 50 km × 50 km resolution grid over Europe. The SOMO35 and SO_2_ fields are used in EVA to estimate acute mortality. The geographical distribution of acute deaths in the base case is displayed in Figure 2 based on both the DEHM and MATCH results. In the Clean Air for Europe Programme of the European Commission (also referred to as the CAFE report [78]) acute mortality was estimated with the Regional Air Pollution and Simulation (RAINS) model to be 21,400 cases in the year 2000 for EU25. A later update by the GAINS model gives ca. 30,000 cases for EU28 [70]. These numbers agree well with our estimates: we get ca. 35,000 (DEHM) and ca. 28,000 (MATCH) as an average for the 2000s and for a slightly larger part of Europe (including 30 countries in total, See S1 in the Supplement Material for population data). 

**Figure 3:**
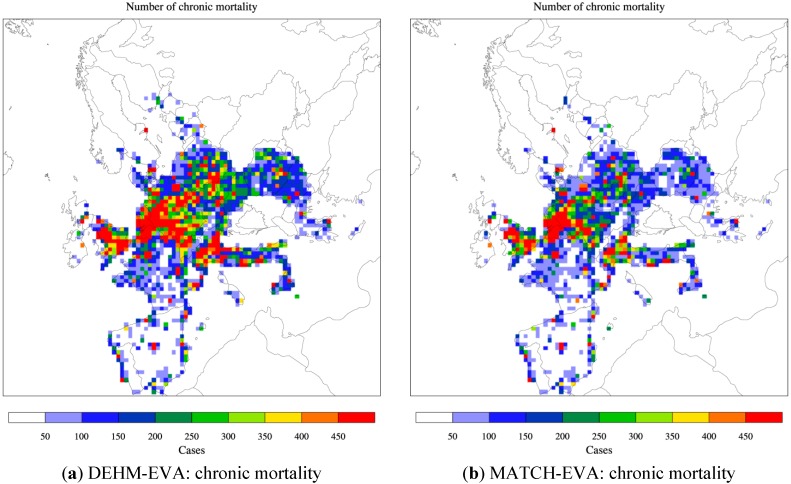
The spatial distribution of chronic mortality across Europe related to PM_2.5_. Based on DEHM (**a**) and MATCH (**b**) simulations with climate, emissions and population data for 2000 (the base “present day” case). The area of a grid cell is 50 km × 50 km = 2500 km^2^. The colour bar refers to number of cases per year per 2500 km^2^. See Figure 5 and Figure 7 for the total number for Europe and the Nordic area.

Similar distributions of chronic deaths are displayed in Figure 3 for the base case. In EVA the so-called chronic mortality is linked to PM_2.5_ pollution and is estimated as the number of years of life lost (YOLL). According to the CAFE report this can be converted to number of premature deaths by dividing YOLL with 10.6 as an average for Europe. The health burden can also be expressed by other measures than YOLLs. In the continuous global burden of disease project the Disability adjusted life years (DALYs) lost (or gained by intervention) has been used as a metric to describe the premature mortality. This metric comprises more than just death, but the weight given to different conditions may be debated. WHO also included annoyance due to exposure to noise, but the weight given to this outcome may vary according to e.g., cultural differences. High numbers of premature deaths are seen in both Figure 2 and Figure 3 in areas with high population densities (e.g., in large cities) and high ozone and/or PM levels. This is e.g., seen in the Benelux region stretching into Germany, in the southern part of England as well as in the Po Valley. For chronic mortality a total of 347,900 premature deaths were estimated in the CAFE report (from YOLL/10.6) for EU25 (due to primary PM_2.5_ and SIA). In this study we estimate between 420.575 (DEHM) and 295.519 (MATCH) premature deaths for the extended area. 

These results were expected from the evaluation of the base case, where DEHM in general overestimates secondary inorganic aerosol, while both models underestimate the primary particles. The spatial distribution for both acute and chronic mortality is similar for the two models, which is due to the common input emissions and population. 

The numbers achieved here also compare well with what was obtained in a previous study [79], where 245,000 premature deaths per year in Europe were estimated for the period 1997–2003 due to SIA exposure on the regional background scale using reanalysis meteorology as input to MATCH. 

**Figure 4:**
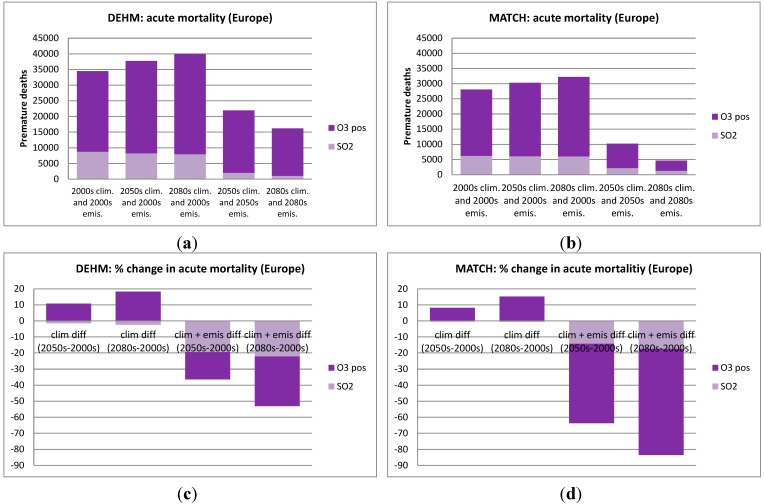
Acute mortality as estimated by DEHM (left) and MATCH (right) for the European region. In (**a**) and (**b**) the first three columns show the number of premature deaths in the 2000s, the 2050s and 2080s, when only changing the climate. The last two columns includes both climate and emissions changes for the 2050s and the 2080s; In (**c**) and (**d**) the change in % relative to the 2000s are displayed.

**Figure 5:**
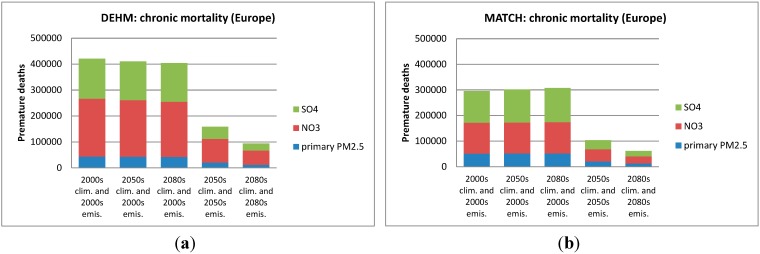

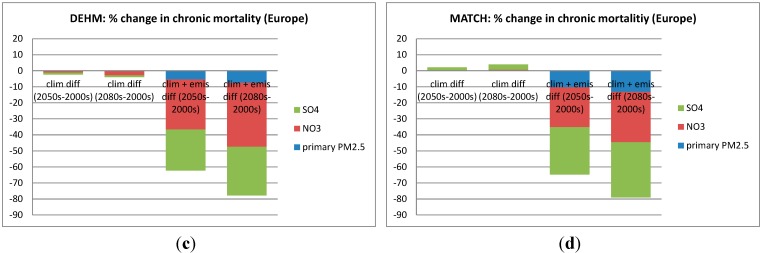
Chronic mortality as estimated by DEHM (left) and MATCH (right) for the European region. In (**a**) and (**b**) the first three columns show the number of premature deaths in the 2000s, the 2050s and 2080s, when only changing the climate. The last two columns includes both climate and emissions changes for the 2050s and the 2080s; In (**c**) and (**d**) the change in % relative to the 2000s are displayed. The impact of the different PM components is also indicated. Note that “SO_4_” here includes the full weight of SO_4_^2−^, H_2_SO_4_, NH_4_SO_4_ and (NH_4_)_2_SO_4_, while “NO_3_” includes the full weight of NO_3_^−^ and NH_4_NO_3_. “primary PM_2.5_” refers to the mass related to primary emitted BC and OC.

**Figure 6:**
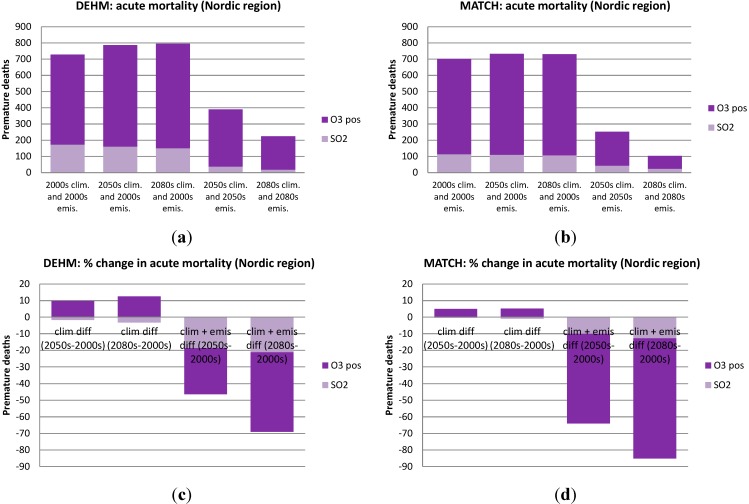
As in Figure 4, but for the *Nordic region* only (Sweden, Finland, Norway and Denmark).

**Figure 7:**
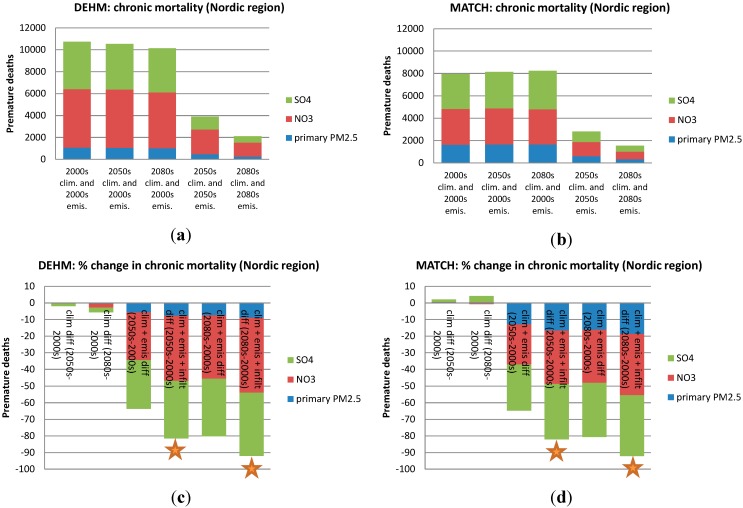
As in Figure 5, but for the *Nordic region* only (Sweden, Finland, Norway and Denmark). Here an estimate of the change in infiltration rate has also been included in the simulations where both climate and emissions are changed (see Section 3.5). These simulations are marked by a star.

In Figure 4, Figure 5, Figure 6 and Figure 7 the numbers of acute and chronic deaths as a total for Europe and the Nordic region alone are shown for the base case and for the future projections. The total columns also indicate the fraction caused by the different chemical components. For chronic mortality the largest difference between the two models is seen to be caused by the difference in the NO_3_ component. The evaluation shows that DEHM overestimates this component in this climate setup. 

For the Nordic region (Figure 6 and Figure 7) the number of acute deaths is in the base case estimated to be 728 (DEHM) and 701 (MATCH). A somewhat larger difference is seen for the chronic deaths with an estimate of 10,743 cases with DEHM and 8134 cases with MATCH. Only few previous studies have been focused on the Nordic region. For acute mortality in the Nordic region, a slightly higher number of cases (around 785–945) were obtained in an earlier study based on MATCH using two different climate realizations [20]. The CAFE estimates are only given for EU member states, whereby only part of the Nordic is covered. Thus, the CAFE estimates for the Nordic region only include Denmark, Sweden and Finland and amount to 432 and 7820 for acute mortality and chronic mortality, respectively. In the present study this would be 623 (DEHM) and 490 (MATCH) for acute mortality and 8905 (DEHM) and 5964 (MATCH) for chronic mortality.

### 3.2. Changes in Climate Alone

In order to quantify the impact of changes in climate alone, the CTM models have been run for the two future periods with current day emissions. Thus, the models are for the period 2050–2059 driven by projections of meteorological data for the 2050s and emissions for year 2000. The same is the case for the period 2080–2089. The population data set for year 2000 is used in all three periods. 

Using the results of both models in the EVA system leads to a general increase in the number of acute deaths across Europe compared to the 2000s. The increase is 8%–11% and 15%–16% towards the 2050s and the 2080s respectively according to the two CTMs (Figure 4c,d). This increase is linked to the well-known climate penalty on ozone mainly in southern Europe discussed in several previous studies [8,9,10,31], which is mainly driven by increased natural emissions of ozone precursors (BVOCs like isoprene) in a warmer climate. A few studies with focus on the US, have also estimated the health impact associated with a climate penalty on ozone and they all found an increase in the O_3_-related acute mortality (see [23] for an overview). 

In EVA the impact of SO_2_ and O_3_ on acute mortality can be analyzed separately; a small decrease is projected due to a slight decrease in SO_2_ concentration in DEHM, while no changes are projected for SO_2_ in MATCH. This can be caused both by the differences between the global and regionally downscaled climate data used by the two models or differences in the climate sensitivity in the models. In the earlier model intercomparison including both models, a more profound increase in the BVOCs emissions was found in DEHM compared to MATCH [10]. The environmental processes driving the BVOC emissions and hence the sensitivity to future changes in climate, land-use and e.g., in atmospheric CO_2_ levels is, however, still not well understood and currently not well described by present day models [72,80,81,82]. 

**Figure 8:**
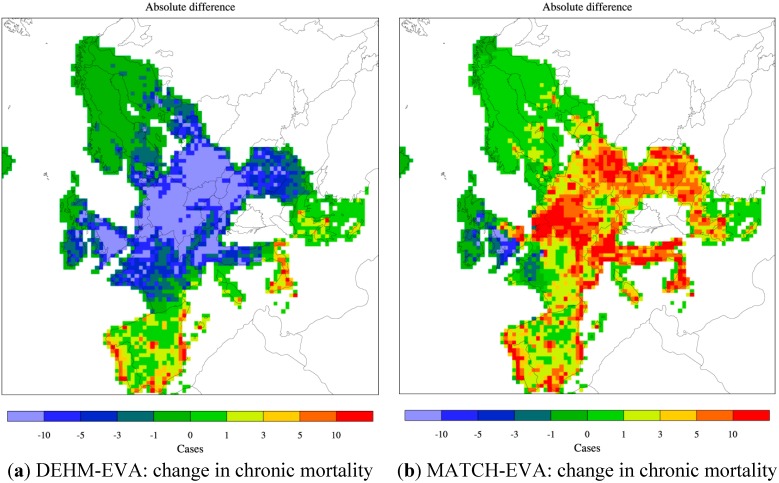

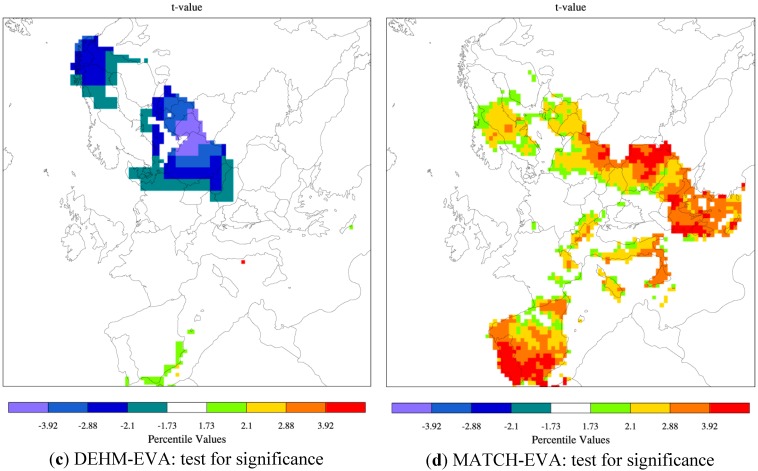
Climate change alone: change (absolute difference) in chronic mortality from present (2000s) to future (2080s) as estimated by DEHM-EVA (**a**) and MATCH-EVA (**b**). The colour bar refers to number of cases per year per 2500 km^2^. The change has been tested for significance and the corresponding *t*-value is given below (**c** and **d**) for each model. Only in areas with a colour different from white the estimated difference is significant. The threshold value for significance is chosen to 10%.

For chronic mortality the sensitivity to climate changes differs between the two models. DEHM points towards a small (2%–4%) decrease in the total number of chronic deaths, while MATCH simulates a similar increase due to climate change alone. The geographic distribution of the changes varies across Europe (Figure 8a,b) and both models agree on an increase in the chronic mortality in southern Europe, but disagree on the sign of the change in central and northern parts of Europe. These changes are all very small. In order to test for statistical significance in the change of the mean value compared to the interannual variability, the student’s t-test was conducted. It shows that the change is only significant in limited areas (the colored areas in Figure 8c,d). The climate change impact on the levels of PM is mainly linked to changes in precipitation (amount and/or frequency of precipitation events), but changes in e.g., solar radiation, temperature and humidity can also effect the gas-aerosol equilibrium and hence the formation of aerosols in the atmosphere [8,19]. 

Both climate models used here project a decrease in precipitation in the southern part of Europe, which could then lead to less washout of PM and hence to the small increase in chronic mortality in this part of Europe seen in both DEHM and MATCH (Figure 8a,b). The current comparison between DEHM and MATCH indicates that the geographical pattern of changes in PM due to climate change is much less robust than for O_3_, and this finding is further supported by previous model studies [8]. 

The change in air pollution related deaths due to climate change in the Nordic region is quite similar to the general results for Europe. The number of acute deaths increases by less than 10% according to both models (see Figure 6c,d). For chronic mortality the number of cases per year increases by a few percent towards the 2080s according to the MATCH model, while the DEHM results gives a slight decrease in the yearly number of cases. Future climate change is expected to decrease the surface level of O_3_ in Northern Europe (e.g., Langner, Engardt and Andersson [41]), and this is also seen here, where the simulations show a decrease in the most Northern parts of the Nordic region. A slight increase is, however, estimated for other parts of the Nordic. A *t*-test shows that the changes in health effects in the Nordic countries due to climate changes are non-significant in comparison to the interannual variability.

### 3.3. Change in Both Climate and Anthropogenic Emissions

**Figure 9:**
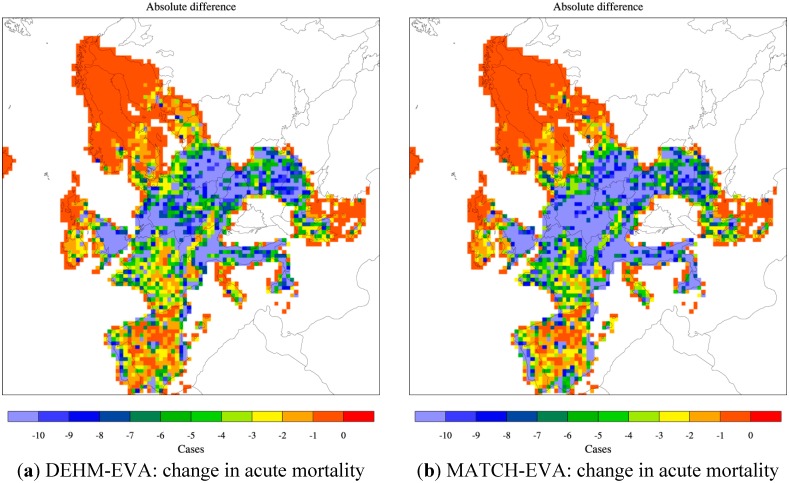

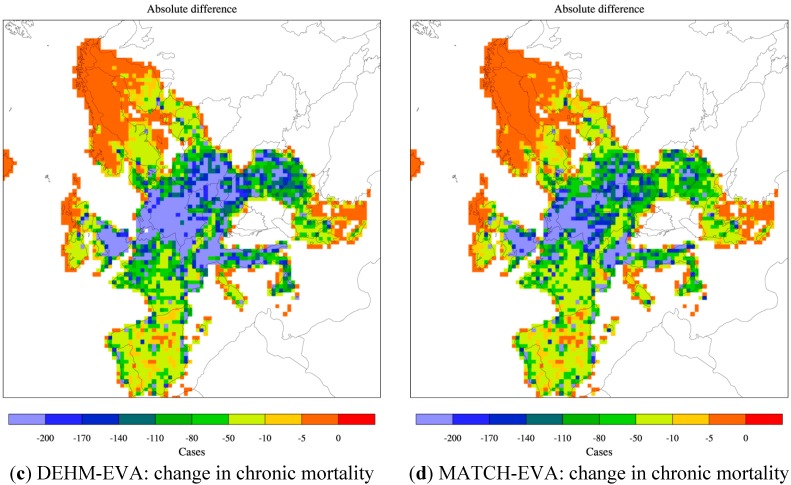
Combined climate and emission changes: change in (absolute difference) acute (**a** and **b**) and chronic mortality (**c** and **d**) from present (2000s) to future (2080s) as estimated by DEHM-EVA (a and c) and MATCH-EVA (b and d). The colour bar refers to number of cases per year per 2500 km^2^.

The combined effect of projected changes in both climate and anthropogenic emissions is simulated by the CTM models using both meteorological data for the 2050s (2080s) and emissions for 2050 (2080). The population data in EVA is still for the year 2000. The applied emission scenario projects a large decrease in the O_3_ precursor NOx (Table 1) and in both models this reduction poses a stronger effect than climate change alone. As seen on Figure 4c the DEHM-EVA system projects a 36% and 53% decrease in the number of yearly acute deaths towards the 2050s and 2080s. For the combined MATCH-EVA setup larger decreases of 64% to 84% in the O_3_-related mortality are simulated. The geographic distribution of the difference in acute mortality between the 2080s and 2000s as estimated by the two models is seen in Figure 9a,b. Largest decreases are seen in the highly populated central part of Europe. The climate penalty on O_3_ in Southern Europe is compensated by emissions changes, which likewise has been shown by similar recent CTM studies [8,41]. At the global scale it has been estimated that potential emissions changes can translate into significant reductions of O_3_-related premature mortality [83]. 

In terms of the impact from PM pollution and chronic mortality, the combined changes in climate and emissions results in a decrease of 62% in the 2050s and 78% in the 2080s compared to present day (2000s) conditions according to DEHM-EVA. MATCH-EVA estimates very similar results: a decrease by 65% in the 2050s and 79% in the 2080s. Further the geographical allocation of the change in mortality is very similar between the models (Figure 9c,d). As for O_3_, the projected decline in emissions results in significant reductions in PM-related health effects across all of Europe. This is in accordance with earlier findings showing that PM_2.5_ levels are even more dominated by the emission signal than O_3_ [7]. The largest decrease in anthropogenic emissions is projected for SO_x_ (see Table 1). As a result sulfate decreases more than the other PM components included here, whereas in both DEHM and MATCH the nitrate part of the total PM_2.5_ mass is increasing in the future. This is in accordance with [8], where the fraction of nitrate is seen to increase when including changed emissions and climate. 

It has not been possible to compare our estimates of premature deaths in Europe against other studies for the future periods in focus here. The most recent assessment with the GAINS model includes for example only changes in emission and only towards the year 2030 [70]. 

The additional scenario where the ammonia emissions are increased by 35% above expected values in 2080 has only been run by the DEHM-EVA setup. Ammonia contributes to the formation of particles through chemical transformation into secondary inorganic aerosols in the atmosphere. By comparing the estimated number of chronic deaths in the standard simulation for 2080s with this new simulation, the effect of the increased ammonia emission is isolated. The difference between the two simulations for the 2080s is a 4% increase in the chronic mortality in Europe related to PM_2.5_. The increase is only statistically significant in areas with a large emission of ammonia, such as Poland and Northern parts of Germany. 

The impact of combined emission and climate changes for mortality in the Nordic region can be seen in Figure 6 and Figure 7. The number of acute deaths is projected to decrease by 69% and 85% towards the 2080s according to the DEHM-EVA and MATCH-EVA models (Figure 6c,d). Chronic mortality for the Nordic region is reduced by ca 80% from the 2000s to the 2080s in both models (Figure 7c,d). 

### 3.4. Change in Population

So far no projection on population has been used. In this section we assess the effect of changing population data by using population data of the year 2000 and 2050 in the EVA system. We do this sensitivity test using the CTM simulations for the 2050s (based on emissions and meteorology of this decade). The geographical distribution of the population change is shown in Figure S2b in the Supplementary Material.

**Figure 10:**
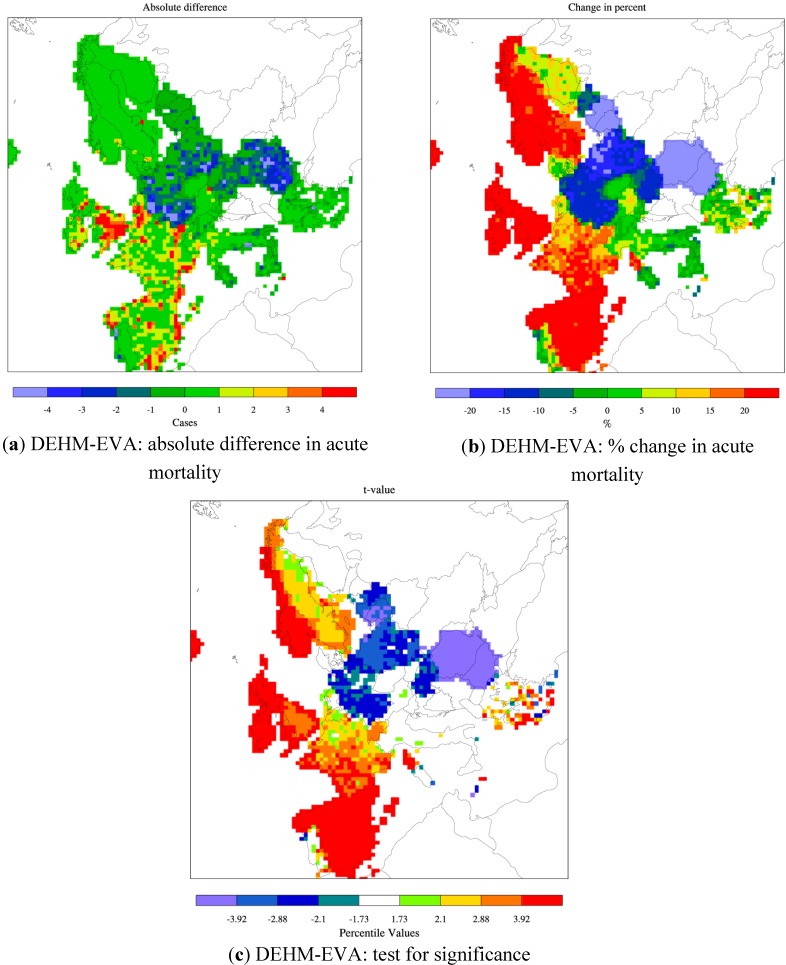
Changes in population: the absolute difference (**a**) and the change in % (**b**) of acute mortality when using the 2050 population data in the assessment for 2050 instead of the population data for 2000. The change has been tested for significance and the corresponding *t*-value is given in (**c**). Only in areas with a colour different from white (in panel c) the estimated difference is significant. Based on the DEHM-EVA results. The threshold value for significance is chosen to 10%.

The number of premature deaths related to chronic mortality is estimated to increase by ca. 13%, while the acute mortality is increasing by ca 3%, when using population data from 2050 instead of the data for year 2000. In the EVA system the exposure-response functions are in many cases age dependent e.g., the chronic mortality related to exposure to PM is only applicable for the population older than 30 years of age. The total European population is projected to increase by ca. 3% from 2000 to 2050. Nevertheless, this increase is not evenly distributed within the applied age classes (see Figure S1a in the Supplementary Material). The population is in general getting older with a dramatic increase (by more than 85 %) in the number of persons above 65 years of age and a decrease (by ca. 12%) in the number of children. This explains why the relative increase in chronic mortality is larger than the relative increase in the total population. The use of the projected population data for 2050 results in large spatial changes in the mortality compared to the case where the data for 2000 is included. This is linked to the facts that the population in some countries is projected to grow in the future (e.g., United Kingdom, Spain and France), while it is projected to decrease in other countries (e.g., Germany, Poland, Romania and Bulgaria), and for some countries the change is very small [57]. These variations are reflected in the spatial distribution of the simulated change in acute mortality (Figure 10). These variations are reflected in the spatial distribution of the simulated change in acute mortality (Figure 10). From this distribution it can be seen that the DEHM-EVA setup indicates a decrease in acute mortality (~10%–15%) in e.g., Germany and Poland. Even larger increases in acute mortality are simulated for countries like United Kingdom and France, where the population is projected to increase.

A few other studies have included projections of the population [23,84], but most assessments of future mortality rates in Europe are based on the present day demography. 

In the Nordic region the fraction of older people is also projected to increase towards 2050 (Figure S1b in the Supplementary Material). Among the Nordic countries, it is in Sweden and Norway that the overall increase in the population is so large that the impact of the population data leads to significant changes in the number of acute premature deaths (Figure 10c). 

### 3.5. Change in the Nordic Building Stock and Infiltration

Finally we have tested if a change in building tightness and hence the infiltration of outside air pollution into buildings will have a significant impact on the assessment of air pollution related health effects in the future. These estimates are calculated by assuming that in Nordic countries the dominant technology in improving energy efficiency is mechanical ventilation, implemented together with heat recovery including efficient filtration of intake air. The fractions from Table 2 are used to scale the estimated chronic mortality for the Nordic region by assuming a relative change in the infiltration rate compared to the base case for the 2000s. It is assumed that the change in infiltration affects the different PM_2.5_ components included here equally. This is commonly assumed in assessments like this, but differences in infiltration rates should be analyzed in more detail in future studies. 

By 2020, the indoor exposure to ambient PM_2.5_ concentration ratio has lowered by 13% in comparison with year 2000 used as baseline here, and by 2050 the reduction has reached 49%. In 2080 the remaining indoor-outdoor exposure ratio is only 35%, indicating that two thirds of the outdoor pollution impacts on indoor exposures have been removed by the building stock. Please note that we are not including any indoor sources to this exposure—we are focusing on the indoor exposure to outdoor air. When translated into PM_2.5_ related premature mortality by the use of DEHM-EVA and MATCH-EVA (Figure 7c,d) this assessment shows that the lower infiltration potentially adds significantly to the reductions in the number of deaths. The results indicate that the reduction in chronic mortality due to combined climate and emissions changes by 2080, could be reached already in 2050 due to the projected change in building stock. 

Indoor-outdoor relationship of PM concentrations are not considered in major health impact studies (e.g., [1]) which is a major shortcoming for estimation of long-term trends in health impacts caused by air pollution. Recent studies have attempted to add considerations for ventilation and indoor sources (e.g., [66]) and impacts on burden of disease [76]. However, such work has not been integrated with long-term climate change projections until now, even though the principles of (nearly) zero energy buildings, optimized ventilation and increased building tightness have been well described as policy targets [60,63]. In for example Finland, requirements for improved filtration of intake air has been required in the building codes in urban areas since 2002. Accounting for the gradual change of building stocks, such regulation needs a few decades to penetrate; after such a delay the impacts of the regulations are likely to be realized in the physical properties of the buildings and should be accounted for in health impact assessments. 

## 4. Conclusions

Our study contributes to improving future health assessments by focusing on sensitivities caused by the choices of assumptions and input data. With an integrated assessment model covering Europe we provide a comprehensive sensitivity study that includes: (a) climate change; (b) projected changes in anthropogenic emissions (c) climate impacts on agricultural emissions; (d) change in demography and (e) change in building structure. All these affects are then evaluated in relation to the health effects in the future. In our study gridded maps of air pollution from two different CTMs (DEHM and MATCH) have been used as input to the Economic Valuation of Air pollution (EVA) system. Based on demographic data and exposure-response functions in EVA the exposure and the related health effects are estimated. Here the focus has been on premature mortality linked to short term exposure to O_3_ and SO_2_ (acute mortality) and the long-term exposure to PM_2.5_ (chronic mortality). Our current estimate for Europe is that chronic mortality is about 10 times higher than acute mortality. In this estimate we include only primary anthropogenic particles (BC/OC) and secondary inorganic aerosols (SIA) in the total PM_2.5_. 

The estimated premature mortality under current day conditions shows a reasonable agreement with other similar assessments for Europe. By using projections of the future development in climate, anthropogenic emissions and demography we find that:

- Climate change alone leads to a small increase (ca. 15%) in the total number of O_3_-related acute premature deaths in Europe towards the 2080s. The two CTMs included in the study disagree on the sensitivity of PM_2.5_ related chronic premature mortality to changes in climate alone: one model projected an increase, and the other a decrease, however, both models agreed on relatively small changes (<5%).
- Expected declines in anthropogenic emissions (>50% for most components in both 2050 and 2080) leads to a large decrease in the surface concentration of O_3_, primary PM and SIA across Europe. The impact from decreased emissions exceeds the impact from climate change alone (in agreement with previous studies). The combined effect of climate change and emission reductions will decrease the premature mortality due to air pollution. The acute mortality is in the present study estimated to decrease by 36%–64% in the 2050s and 53%–84% in the 2080s. For PM-related chronic mortality the decrease is 62%–65% and almost 80% for the two future periods.
- A part from the well-known climate penalty on ozone, we also include a recently quantified climate penalty on agricultural ammonia emissions. The simulations for the 2080s show that increased ammonia emissions mainly affect PM_2.5._ concentrations in regions with high ammonia emissions (e.g., Germany, Poland, Netherlands and Belgium). Europe-wide this effect alone will increase the chronic mortality by 4%.
- The assessment also shows sensitivity to the used population distribution and age structure towards 2050. The expected change in population size and age during this 50 year period is so substantial that it has a notable impact (~13%) on the projected health outcome related to air pollution in some areas in Europe.
- For the Nordic region we have projected how the infiltration rate into buildings will change in the future due to improvement of energy efficiency of building. By assuming a relative change in the infiltration rate compared to current day and a building stock renewal rate of 2% per year, projections are made for the future infiltration. Our assessment shows that by including this parameter the estimated decrease in PM_2.5_-related premature deaths could be ca. 80% in the 2050s relative to current day instead of the ca. 62%–65% as caused by climate and emissions changes alone. For the 2080s the decrease is estimated to be ca. 90%, while climate and emissions changes alone gave ca. 80%.

We emphasize that assessments like the present study are associated with large uncertainties and the projections should be used to illustrate a possible tendency in the future under the sensitivity to the input data. The largest limitation in this study is the lack of secondary organic aerosols (SOA) in the models applied. The current day PM-related health effects are thereby underestimated (~20%) and the sensitivity to future changes in both climate and emissions will also be underestimated. More knowledge and better descriptions of SOA in models are necessary in order to limit the uncertainty of this kind of assessment. Especially, more research on biogenic VOCs and potential future changes with e.g., climate, land-use and CO_2_ is crucial for better projections of the climate penalty on both O_3_ and PM_2.5_. The climate penalty on natural and e.g., agricultural emissions can counteract the effect of current air quality regulations and therefore the impact of climate change needs to be included in health assessments for the future. A better quantification of component-specific toxicity and appropriate response functions are also of high importance. Combined with a higher spatial resolution, such functions could make it possible to evaluate the effect of regulation of e.g., traffic with less uncertainty. Most assessments use present day demography also for future projections. Our results show that this will introduce a systematic bias to the estimates. This underlines the importance of including a valid population data set and/or more than one projection in order to get a better estimate of the future changes. Energy savings have been high on the political agenda in the EU for many years, which impacts the building stock and the infiltration rate. The effect of these initiatives on the premature mortality has never been assessed, but here we have extended the integrated model system to include an extra term unfolding the impact of changed infiltration rates in the Nordic region. This shows that the building stock and its improvement, is an important factor that ought to be included in long term projections of premature deaths due to of PM_2.5_ exposure. Our approach should nevertheless be elaborated and this initial model for accounting for the changes in the building stock can be improved, when more information becomes available regarding the European policies on buildings.

Finally, the inter-model differences seen here underlines the importance of using an ensemble of both climate and chemistry transport models in order to span the range of possible developments in climate, anthropogenic emissions and air quality. 

## Supplementary Files

Supplementary File 1
