# Supplementary material for: Future Premature Mortality Due to O3, Secondary Inorganic Aerosols and Primary PM in Europe — Sensitivity to Changes in Climate, Anthropogenic Emissions, Population and Building Stock
Source: Int J Environ Res Public Health. 2015 Mar 4;12(3):2837–69. doi: 10.3390/ijerph120302837 (PMC4377936; doi:10.3390/ijerph120302837)
Supplement: Supplementary File 1 [file ijerph-12-02837-s001.pdf]

Supplementary Information

Future Premature Mortality due to O_3, Secondary Inorganic Aerosols and Primary PM in Europe — Sensitivity to Changes in Climate, Anthropogenic Emissions, Population and Building stock

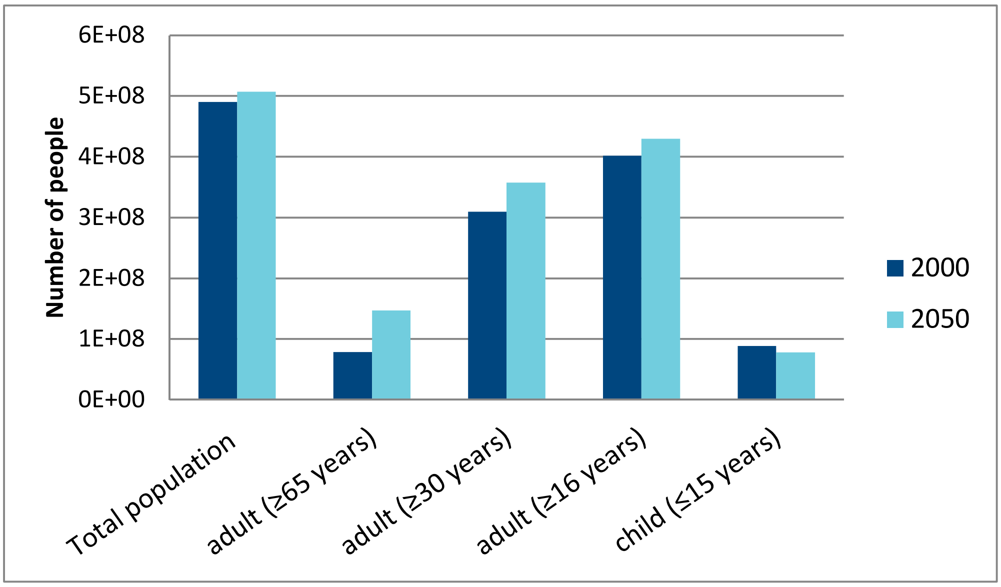

(**a**)

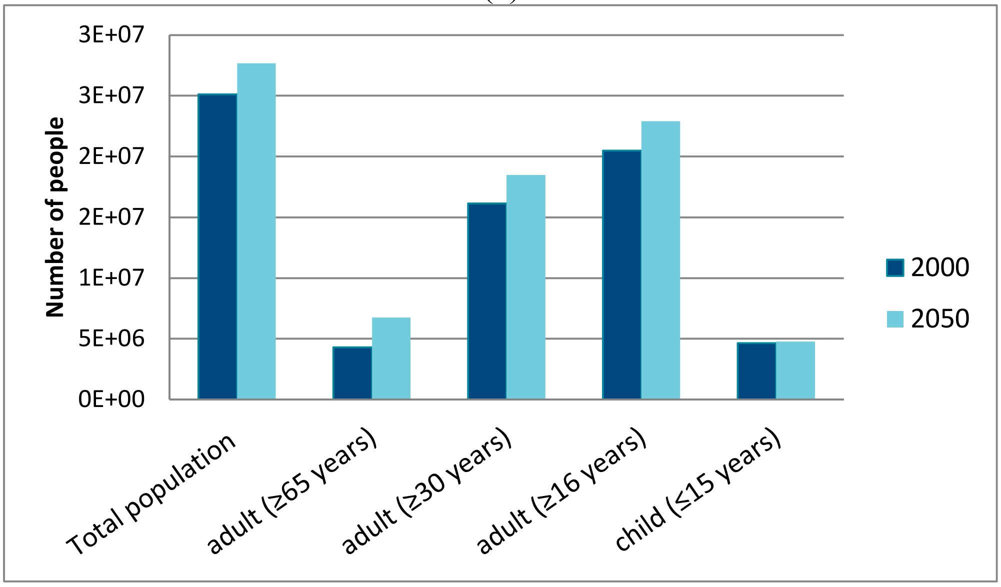

(**b**)

Figure S1. The applied population data sets for 2000 and 2050 (including the division into different age classes). (**a**) is the total numbers for Europe and (**b**) is the total numbers for the Nordic region (Sweden, Norway, Finland and Denmark).

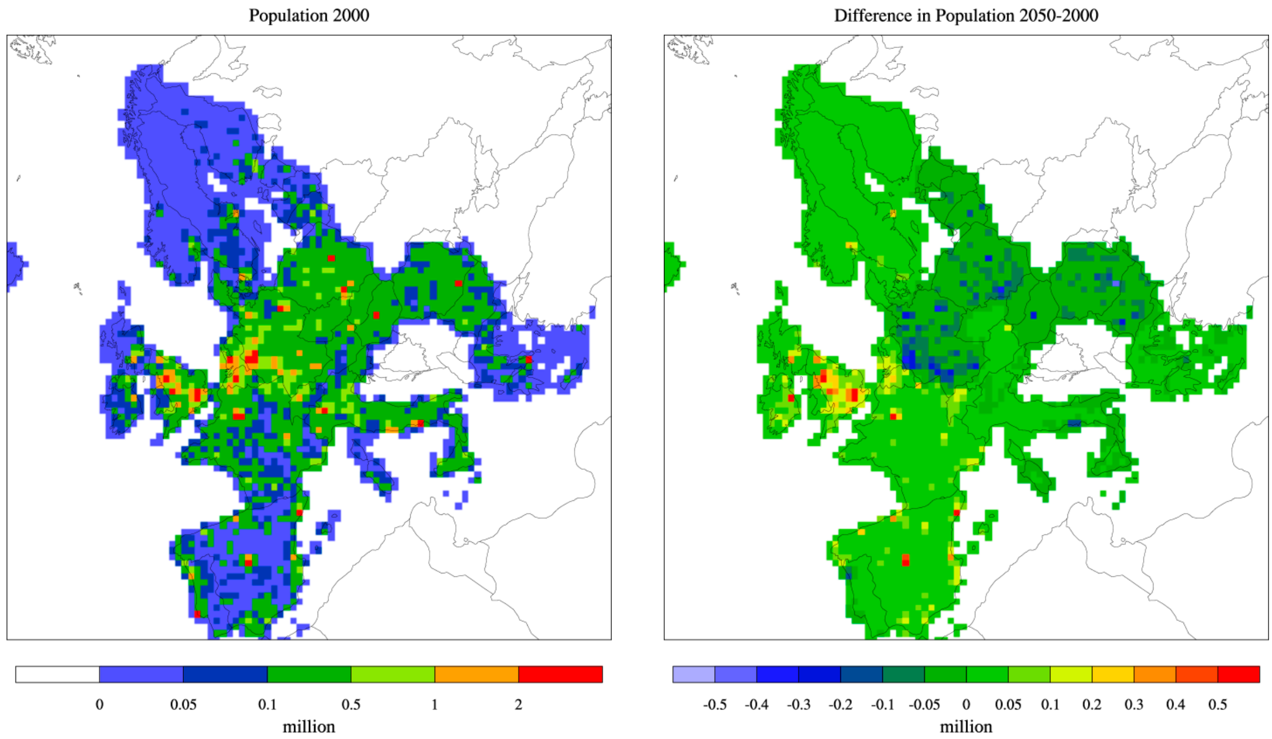

(**a**) (**b**)

Figure S2. The applied distribution of the population across Europe for year 2000 (**a**). The absolute difference between the population data for 2050 and 2000 is shown in (**b**). Based on data and projections for 30 countries. From: http://www.integrated-assessment.eu/.

© 2015 by the authors; licensee MDPI, Basel, Switzerland. This article is an open access article distributed under the terms and conditions of the Creative Commons Attribution license (http://creativecommons.org/licenses/by/4.0/).
